# Matrilin-2 Is a Widely Distributed Extracellular Matrix Protein and a Potential Biomarker in the Early Stage of Osteoarthritis in Articular Cartilage

**DOI:** 10.1155/2014/986127

**Published:** 2014-03-11

**Authors:** Shukun Zhang, Jinwu Peng, Yan Guo, Sara Javidiparsijani, Guirong Wang, Yichun Wang, Honggang Liu, Jingshi Liu, Junming Luo

**Affiliations:** ^1^Department of pathology, Beijing Tongren Hospital, Capital Medical University, No. 1 Dongjiaominxiang Street, Beijing 100730, China; ^2^Department of Pathology, Qinghai Provincial People's Hospital, 2 Gonghe Road, Xining, Qinghai 810007, China; ^3^Department of Pathology, The Affiliated Xiangya Hospital of Xiangya School Medical School, Central South University, Changsha 410013, China; ^4^Department of Surgery, SUNY Upstate Medical University, 750 E. Adams Street, Syracuse, NY 13210, USA; ^5^Department of Anesthesiology, Hunan Provincial Tumor Hospital, The Affiliated Tumor Hospital of Xiangya School Medical School of Central South University, 283 Tongzipo Road, Changsha, Hunan 410013, China

## Abstract

In this study, we first generated and characterized a polyclonal antibody against unique domain of matrlin-2 and then used this specific antibody to assess the expression pattern of matrilin-2 by immunohistochemistry. We found that marilin-2 is widely distributed in the connective tissues of many mouse tissues including heart, colon, penis, esophagus, lung, kidney, tracheal cartilage, developmental bone, and adult bone. The expression level of matrilin-2 was remarkably increased in the tissues of osteoarthritis developmental articular cartilage, compared to normal healthy tissues. Furthermore, we determined matrilin-2 expression in specific epithelial cells in stomach and ductal epithelial cells of salivary gland. In other tissues, the positive signals were mainly located around cardiac muscle cells and Purkinje fibers in the heart; corpus spongiosum in the penis; submucosa in the colon and esophagus; extracellular matrix of cartilage in the tracheal cartilage; and, glomerulus, the basement membrane of distal convoluted tubule and renal matrix in kidney. These observations indicated that the distribution pattern of matrilin-2 is heterogeneous in each tissue. Matrilin-2 may play an important role in the communication of matrix to matrix and matrix to cells and will be used as a potential biomarker in the early stage of osteoarthritis of articular cartilage.

## 1. Introduction

Extracellular matrix (ECM) is composed of a large number of secretary multiple domain proteins, which form a filamentous network to connect cell surface and other ECM molecules. ECM proteins mediate cell-matrix and matrix-matrix communication and thereby determine the histoarchitecture specific to every organ and provide cells with crucial information on migration, adhesion, and differentiation [1–4]. The multiple domain proteins share homologous modules that consist of specific protein domains derived from common evolutional origin which form oligomer with itself or other proteins and consist of multiprotein complexes [1, 5–7]. Matrilin is a novel filamentous-forming adapter extracellular matrix protein family, which can form collagen-dependent and collagen-independent network and is involved in the development and homeostasis of network of extracellular matrix [1, 4–6, 8]. There are four members in this family, which are named matrilins 1, 2, 3, and 4 [1, 5, 9]. Matrilin-2 is the largest member of this family, which shares homologous modules with the other three members [1, 5, 6, 9]. They all contain von Willebrand factor A (vWFA) domains, epithelial growth factor (EGF) like repeats, and a series of heptad repeats at their C-terminal coiled-coil domain, which is a nucleation site for oligomerization [1, 9–14]. Although there are several reports about the distribution of matrilin-2 by immunohistochemistry in some tissues [7, 10, 15–19], a complete distribution pattern of this protein has not been established because of the limitation of the antibodies used in previous works. Immunohistochemistry is a useful tool to determine the localization of the antigen. However, the primary antibody is a critical factor for the liability of the results of this method. Because vWFA domain and EGF domain of matrilin family are ubiquitous [5, 6], cross reaction with other proteins can occur when matrilin-2 antibody is raised from whole-length matrilin-2.

Although the knowledge about matrilin-2 functions is accumulating, it still limits at the present time. Matrilin-2 is believed to be a novel family member of filament-forming oligomeric adapter proteins that are involved in the development and homeostasis of the extracellular matrix network [1, 5–7, 9, 20]. Matrilin-2 serves as one of the major components of basement membranes and a putative adaptor molecule of extracellular matrix, which can form both collagen-dependent and collagen-independent filamentous network [1, 5–10, 13, 20], and is involved in the reorganization of tissue architecture. During liver cirrhosis, hepatocellular carcinoma [17, 21] and sporadic pilocytic astrocytoma [19], the expression level of matrilin-2 was elevated. In addition, matrilin-2 was found to be involved in the balance of keratinocyte and fibroblasts in response to wounding [22] and participated in peripheral nerve regeneration [18], downregulated in early phase of muscle injury, and then increased in its late phase [23].

There are seven putative Smad-binding sites within human matrilin-2 promoter and exon I [22]. Matrilin-2 gene-deficient mice showed no gross abnormalities during embryonic or adult development with normal lifespan [24]. Matrilin-2 consists of a large filamentous network in the body, which acts as an adapter molecule connecting other proteins and proteoglycans in the extracellular matrix and plays an important role in the communication or balance between the extracellular matrix and epithelial cells. The detailed information about the distribution pattern of matrilin-2 in tissues and organs would provide more clues to its biological functions in each individual tissue.

Matrilin-2 contains a unique domain between the second vWFA domain and the C-terminal coiled-coil domain with no sequence homology of other family members and known proteins [1, 9, 13, 20]. To raise matrilin-2-specific antibody, we synthesized peptide sequence corresponding to the C-terminal of the unique region of mouse matrilin-2. Then we raised matrilin-2-specific antibody in the study. We found the antibody raised in this study specifically recognized the unique domain (both long and short forms) of matrilin-2, but no cross reactivity with recombinant matrilin-1 and matrilin-3. Immunohistochemical results of the antibody showed that matrilin-2 is widely distributed in skeletal tissues, dense and loose connective tissues, and some specialized epithelia in mice. These distribution patterns in different tissues may hint tissue heterogeneity.

## 2. Materials and Methods

### 2.1. Animal Tissue and Human Osteoarthritis Tissue Preparation

#### 2.1.1. Nondecalcified Soft Tissues

Newborn C57BL/6 and healthy adult C57BL/6 (6-week-old) mice were anesthetized and then sacrificed with an intraperitoneal injection of phenobarbital. Various mouse tissues including lung, brain, tongue, larynx, pharynx, salivary gland, esophagus, stomach, small intestine, large intestine, lymph node, liver, heart, pituitary, thyroid and parathyroid gland, ovary, oviduct, vagina, prostate, epididymis, spleen, kidney, skin, ureter, and testis were harvested and fixed in 10% PBS-buffered formalin. These fixed materials were routinely processed and embedded in paraffin. Serial sections at thickness of 5 *μ*m were prepared and collected on positively charged glass slides (Superfrost Plus, Fisher Scientific). The sections were dried on a hot plate to increase adherence to the slides.

#### 2.1.2. Decalcified Tissues

Knee joins fromsixadult C57BL/6 mice and three nine-month-old male adult Duncan-Hartley guinea pigs with osteoarthritis developments were harvested and decalcified by 10% EDTA solution. These treated materials were processed and embedded in paraffin. Longitudinal serial sections of bone at thickness of 5 *μ*m were prepared as above.

#### 2.1.3. Nondecalcified Bone Tissues and Goldner Trichrome Stain

Tibias tissues from three nine-month-old male adult Duncan-Hartley guinea pigs with osteoarthritis development were fixed in 70% ethanol and then processed for methyl methacrylate embedding. 5 *μ*m serial sections were stained with Goldner trichrome stain.

#### 2.1.4. Human Osteoarthritis Tissues

The human osteoarthritis tissues were collected from clinic knee arthroplasty. The samples were decalcified and embedded in paraffin, and 5 *μ*m sections were prepared.

#### 2.1.5. Antibody Preparation

A peptide SRSTQKLFHSTKSSGNPLEE corresponding to C-terminal of the unique domain of mouse matrilin-2 was synthesized. The antiserum was raised in a New Zealand white rabbit by standard methods. IgG fraction was isolated from serum with a protein A-Sepharose column (Pharmacia Amersham Biotech, Piscataway, NJ) on a Pharmacia fast-performance liquid chromatography system. The purification procedure was described in detail elsewhere [25].

#### 2.1.6. Recombinant Constructs and Western Blot Analysis

**Figure 1:**
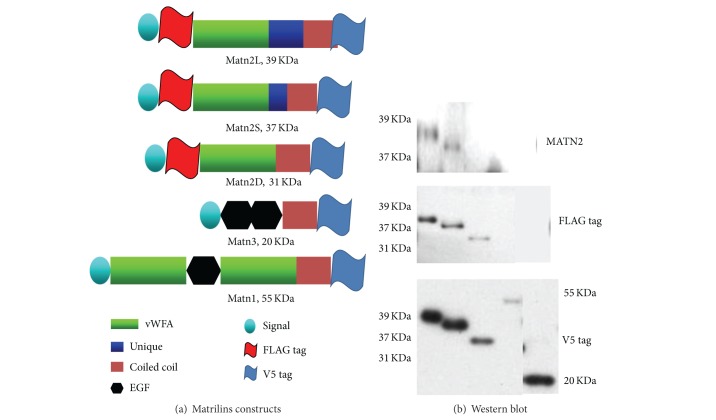
Schematic drawings of the domain structure of recombinant matrilins variants (a) and analysis recombinant matrilin products (b). The recombinant proteins of matrilins were separated on a 4–15% gel, blotted to a membrane, and incubated with antiserum matrilin-2 antibody (upper pattern, both long and short forms of matrilin-2); FLAG tag ((b) middle pattern, long, short, and deleted matrilin-2); V5 tag (bottom pattern, all forms of matrilin-2, matrilin-1, and matrilin-3). The Western blot results showed our antibody can detect two different isoforms of matrilin-2 ((b) lanes 1 and 2 upper). If genetic engineering deletion unique domain mutant of mini matrilin-2, the recombinant product would not be detectable by our matrilin-2 antibody ((b) lane 3 upper); this product can be recognized by tags ((b) FLAG tag, lane 3 middle, and V5 tag, lane 3 bottom). This antibody is no cross reaction with other matrilins, such as matrilin-1 ((b) lane 4) and matrilin-3 ((b) lane 5).

To determine the specification of this antibody raised in this study, we prepared three recombinant constructs including mini-mouse matrilin-2, mini-matrilin-3, and matrilin-1 as described previously [8, 20, 26]. Briefly, transcripts encoding the two isoforms of matrilin-2 were cloned by RT-PCR from mRNA isolated from the rib cartilage of C57BL/6 newborn mice. In the physiological condition, there are two forms of matrilin-2. As shown in Figure 1(a), a miniversion of the transcript containing vWFA2, the unique domain, and the coiled coil was cloned into an expression vector pcDNA3.1/v5-His: matrilin-2L (long) and matrilin-2S (short). In addition, deletion unique domain mutant of mini matrilin-2 was linked by overlapping PCR with described primer sets matrilin-2D (deleted). In addition, a genetic engineering FLAG tag was introduced into all of mini-matrilin-2 cDNA constructs, which would allow us to identify the recombinant protein. Therefore, all of the matrilin-2 constructs contained both FLAG tag and V5 tag, but the matrilin-1 and matrilin-3 constructs only contain V5 tag (Figure 1(a)). The sequences of constructs were confirmed by DNA sequencing. Plasmids containing mini-matrilin-2, matrilin-1, and mini-matrilin-3 were transfected into COS-1 cell as in the statement using Lipofectamine 2000 (Life Technologies, Carlsbad, CA) according to manufacturer's instructions. Seventy-two hours after transfection, the conditioned media were collected for Western blotting analysis.

For the cell culture media, 5 *μ*L conditioned media were mixed with 5 *μ*L of 2x SDS containing 5% *β*-mercaptoethanol reducing gel loading buffer. After boiling for 10 min, samples were loaded onto 4–15% gradient gels (Bio-Rad). After electrophoresis, proteins were transferred onto Immobilon-polyvinylidene difluoride membrane (Millipore). The blots were blocked with 5% nonfat milk (Bio-Rad). Our anti-matrilin-2 peptide polyclonal antibody (1 : 5000), the monoclonal antibody against V5 tag (Invitrogen, diluted 1 : 5000), and polyclonal against FLAG tag (Affinity BioReagents, diluted 1 : 1000) were used as primary antibodies, respectively. The secondary antibodies were horseradish peroxidase-conjugated goat anti-mouse or goat anti-rabbit IgG (H + L) (Bio-Rad, diluted 1 : 5000). Visualization of immunoreactive proteins was achieved using the ECL Western blotting detection reagents (Amersham Pharmacia Biotech) and then exposing the membrane to Kodak X-Omat AR film. The molecular weights of the immunoreactive proteins were determined with two sets of protein markers.

For the newborn and adult mice knee joint, tissue extracts were prepared as described [27]. In brief, the tissues were homogenized thoroughly using a tissue homogenizer in buffered sucrose containing 0.32 M sucrose, 4 mM HEPES, pH 7.3, 1 mM MgCl_2_, 0.5 mM CaCl_2_, 10 mM NaF, and 1 mM Na_3_VO_4_ supplemented with protease inhibitors. Equal amounts (200 *μ*g/well) of proteins were loaded in 4–15% SDS-PAGE gel and blotted on polyvinylidene fluoride membranes using wet transfer system. The blot was incubated overnight at 4°C with our peptide antibody (1 : 5000) and anti-*β*-Actin (1 : 10,000, Sigma, St. Louis, MO). Western blot analysis for the expression of matrilin-2 was carried out as above.

### 2.2. Quantitative Real-Time PCR

Total RNAs were extracted from six newborn and six healthy adult C57BL/6 mice knee joints using RNeasy Kit (Qiagen, Valencia, CA) according to the manufacturer's instructions. For quantitative real-time PCR, the experiment was performed using QuantiTect SYBR green PCR Kit (Qiagen, Valencia, CA) with DNA Engine Opicon 2 Continuous Fluorescence Detection System (MJ Research, Waltham, MA). Primers used in amplification of target genes mRNA are as follows: matrilin-2 (forward, 5′-TGCCTCTGAGCCCATTGACAAG-3′; reverse, 5′-TATGTTGCACTGTTGGCTGGT-3′); 18S RNA (forward, 5′-CGGCTACCACATCCAAGGAA-3′; reverse, 5′-GCTGGAATTACCGCGGCT-3′). The 18S RNA was amplified at the same time and used as an internal control. The matrilin-2 mRNA level was normalized to housekeeping gene 18S RNA levels. The relative value of matrilin-2 mRNA was measured and calculated by computer software (PE ABI, Foster City, CA). The data were presented as mean ± SEM for six samples and analyzed using two-way analysis of variance. The level of matrilin-2 in newborn and adult mice knee joints was designated as 1. Statistical significance was taken at *P* values less than 0.05 (*P* < 0.05).

### 2.3. Immunohistochemistry

Immunohistochemistry was carried out using the Avidin-Biotin Complex (ABC) methods (Vector Labs). Representative sections were deparaffinized and rehydrated through conventional methods. Endogenous peroxidase was blocked by treating the sections with 3% hydrogen peroxide in methanol for 30 min. Digested by bovine testicular hyaluoidase [28] (4000 U/mL in PBS; Sigma, St. Louis, MO) for 30 minutes at 37°C. Nonspecific protein binding was blocked by incubation with 10% normal goat serum. The sections were incubated in a polyclonal rabbit antibody against our matrilin-2 unique domain-specific antibody (1 : 5000) at 4°C overnight. Thereafter, the sections were treated sequentially with biotinylated goat anti-rabbit IgG (Vectastain Elite, Vector Labs) and avidin-enzyme complex (Vectastain Elite, Vector Labs). These treatments were followed by standardized development in 3,3′-diamino b'nzidine (DAB; Zymed). The sections were counterstained with Harris modified hematoxylin (Fisher). In order to investigate the specificity of our peptide antibody, preimmunized serum from the same rabbit and 0.01 M PBS service as negative control.

## 3. Results

### 3.1. Our Antibody Is Specific Recognized Unique Domain of Matrilin-2

In order to investigate the distribution pattern of matrilin-2, a primary antibody was raised in this study. We synthesized a peptide corresponding to the C-terminal of the unique region of mouse matrilin-2. The unique domain is matrilin-2 specific, which does not exist in other matrilins and any known proteins. To ensure the specificity of this antibody, we use matrilin-2L, matrilin-2S, matrilin-2D, matrilin-1, and matrilin-3 transfected into cos-1 cell line. The Western blot results showed the antibody only recognizes expressed proteins from two isoforms of matrilin-2 which contains a unique domain (Figure 1(b), lanes 1 and 2 upper). The expressed protein from deletion mutant without the unique domain of mini-matrilin-2 can only be recognized by tags (Figure 1(b), FLAG tag, lane 3 middle, and V5 tag, lane 3 bottom) but cannot be detectable by the antibody raised in the study (Figure 1(b), lane 3 upper). Importantly, there was no cross reaction with other matrilins, that is, matrilin-1 (Figure 1(b), lane 4 upper) and matrilin-3 (Figure 1(b), lane 5 upper). These results showed that the raised antibody specifically recognized the unique region of matrilin-2 and no cross reaction with matrilin-1 and matrilin-3.

#### 3.1.1. Matrilin-2 Is Widely Expressed in Mouse Dense Connective Tissues (DCT), Loose Connective Tissues (LCT), and Some Specific Epithelial Cells

**Figure 2:**
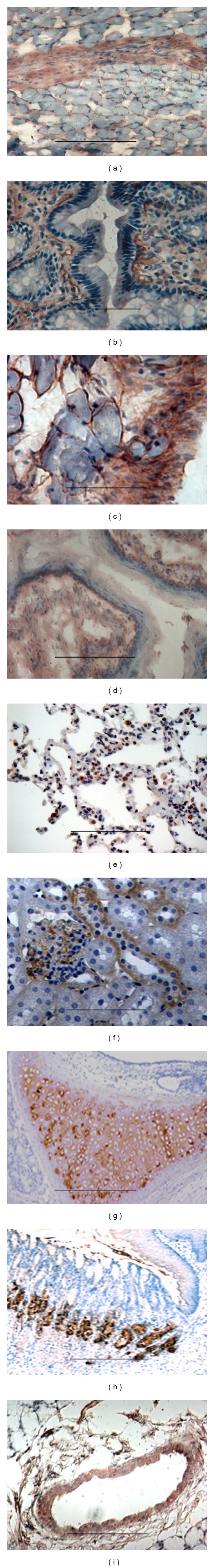
Matrilin-2 expression in mouse dense connective tissues (DCT), loose connective tissues (LCT), and some specific epithelial cells: immunohistochemistry was performed by our unique domain-specific antibody. The localization of matrilin-2 protein was found in DCT, LCT, and some specific epithelial cells of a lot of organs. Immunostaining signal at least was detected in connective tissues of heart (a), colon (b), penis (c), esophagus (d), lung (e), kidney (f), and tracheal cartilage (g). We also found some specific epithelial cells strongly expressing matrilin-2 such as stomach (h) and duct epithelial cells of salivary gland (i). Bar, 200 *μ*m.

Our previous experiments and other studies have shown that matrilin-2 mRNA is widely distributed in several tested tissues by RT-PCR and Northern blot [7, 10, 20]. Here, the expression of matrilin-2 in a variety of adult mouse tissues was investigated by immunohistochemistry using our antibody raised in this study. We found that matrilin-2 protein expression in DCT, LCT, and some specific epithelial cells of a lot of organs (Figure 2). Immunostaining signals were detected in connective tissues of heart (Figure 2(a)), colon (Figure 2(b)), penis (Figure 2(c)), esophagus (Figure 2(d)), lung (Figure 2(e)), kidney (Figure 2(f)), and tracheal cartilage (Figure 2(g)). We also found some specific epithelial cells with strong expression of matrilin-2 such as stomach (Figure 2(h)) and duct epithelial cells of salivary gland (Figure 2(i)). In the heart, the positive signal was found around cardiac muscle and Purkinje fiber (Figure 2(a)); in the penis, the immunoreactive region is located around corpus sponiosm (Figure 2(c)); in the colon (Figure 2(b)) and esophagus (Figure 2(d)), matrilin-2 is strongly expressed in connective tissue of submucosa; in the lung, there are some positive cells in the space of alveoli (Figure 2(e)); in the tracheal cartilage, matrilin-2 expression is located in extracellular matrix of cartilage (Figure 2(g)); and in kidney, a positive signal is mainly shown in glomerulus, the basement side of distal convoluted tubule and renal matrix (Figure 2(f)).

#### 3.1.2. Matrilin-2 Is Inherent Expression in Cartilage and Bone

**Figure 3:**
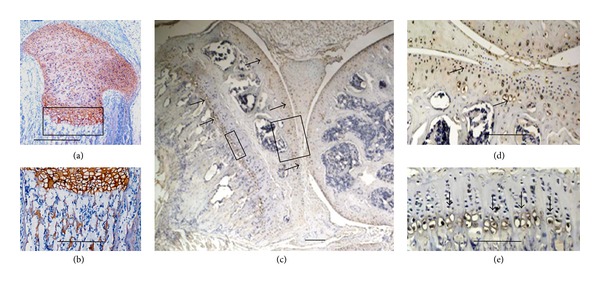
Inherent expression of matrilin-2 in cartilage and bone. Newborn and adult mouse tibiae were investigated by immunohistochemistry. The positive signals localized at the cartilage of newborn mouse tibia including resting, proliferating, hypertrophic zone, and perichondrium, periosteum, new bone marrow, and ligaments (a and b). The strongest signal localized in hypertrophic chondrocytes. In the adult mice, matrilin-2 expression level is much decreased. The positive signal mainly located in articular cartilage and hypertrophic chondrocytes (c, d, and e). There are no positive signals detectable in other zones of cartilage and bone marrow. Bar, 350 *μ*m in (a); 100 *μ*m in (b), (d), and (e); 1 mm in (c).

Matrilins are first isolated from cartilage and are the inherent molecules of skeletal tissues [1, 29]. During embryogenesis and lifespan, all matrilins are expressed in skeletal tissues. The cartilage is the most matrilin abundant tissue. The entire four family members of matrilins are expressed in cartilage. From our immunohistochemistry results, matrilin-2 immunostaining signal at least located the cartilage of newborn mouse tibia including resting, proliferating, hypertrophic zone, and perichondrium, periosteum, bone marrow, and ligaments (Figures 3(a) and 3(b)). The strongest signal localized in hypertrophic chondrocytes. In the adult mice, the expression level of matrilin-2 is dramatically decreased. The positive signal mainly located in surface of the joint of articular cartilage and hypertrophic chondrocytes. There are no detectable positive signals detectable in other zones of cartilage and bone marrow. Matrilin-2 may have an age-dependent expression in skeleton system.

**Figure 4:**
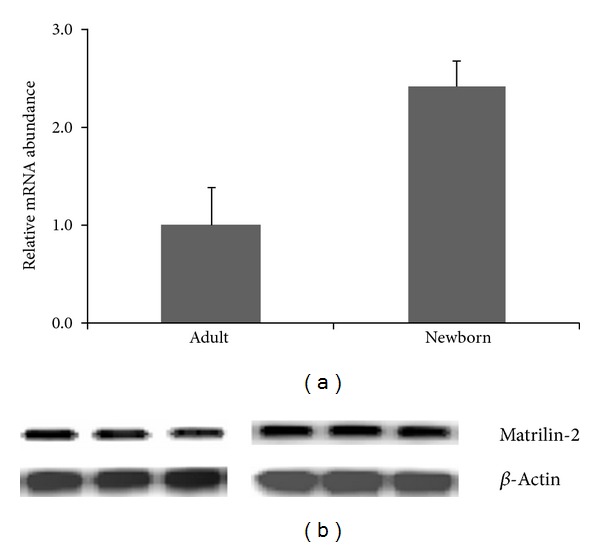
Comparisons the matrilin-2 mRNA and protein expression between newborn and adult mouse knee. Newborn and adult mouse knee were investigated by quantitative real-time RT-PCR (a) and Western blot (b). There is plenty of matrilin-2 mRNA in newborn mouse knee; the level of mRNA is significantly decreased (*P* < 0.001) in the adult mouse knee (a). By Western blot (b), consistent with quantitative real-time RT-PCR (a) and immunohistochemistry results (Figure 3), the protein level of matrilin-2 is significantly decreased in the adult mouse knee (b).

By quantitative real-time RT-PCR, we determine the relative mRNA abundance from 6 newborn and 6 adult mice knee joints. In the adult mice, the relative mRNA abundance of matrilin-2 is 1.017 ± 0.354 and 2.604 ± 0.196, in the adult and newborn mice, respectively. The level of mRNA expression is significantly decreased (*P* < 0.001) in the adult mice compared to the newborn mice (Figure 4(a)). Furthermore, we analyzed matrilin-2 protein expression in the knee of 3 newborn and 3 adult mice by Western blot analysis. The protein level of matrilin-2 is also significantly decreased in the adult mice compared to the newborn mice (Figure 4(b)). These results are consistent with the observation by quantitative real-time RT-PCR and immunohistochemistry.

#### 3.1.3. Matrilin-2 May Be Used as an Early Biomarker of Osteoarthritis

**Figure 5:**
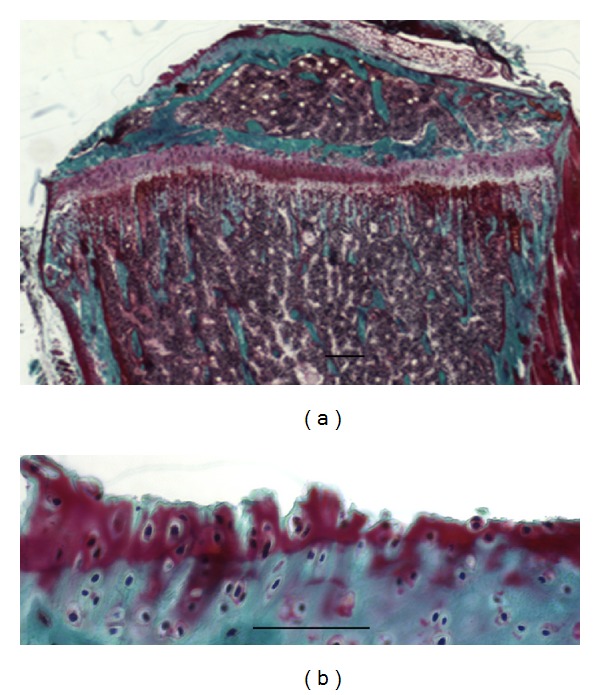
Goldner staining showed damage of articular cartilage in an osteoarthritis (OA) developmental animal model of Duncan-Hartley guinea pigs. OA developmental animal model of Duncan-Hartley guinea pigs tibiae was investigated by Goldner staining. The Goldner staining showed there was damage in articular cartilage. (a) is low magnification; (b) is higher magnification. Bar, 1.5 mm in (a); 100 *μ*m in (b).

**Figure 6:**
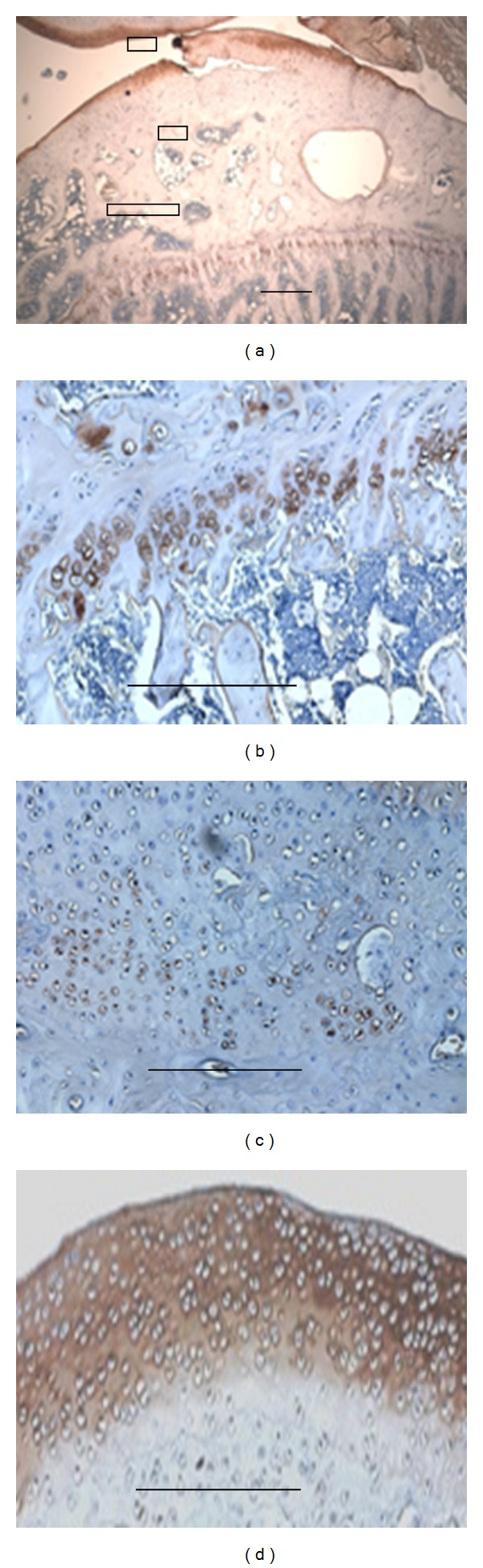
Matrilin-2 expression in Duncan-Hartley guinea pigs tibiae. OA developmental animal model of Duncan-Hartley guinea pigs tibiae had been investigated by immunohistochemistry. The positive signal mainly located in articular cartilage and hypertrophic chondrocytes (a–d). There are no positive signals detectable in other zones of cartilage and bone marrow. Bar, 1 mm in (a); 100 *μ*m in (b), (c), and (d).

**Figure 7:**
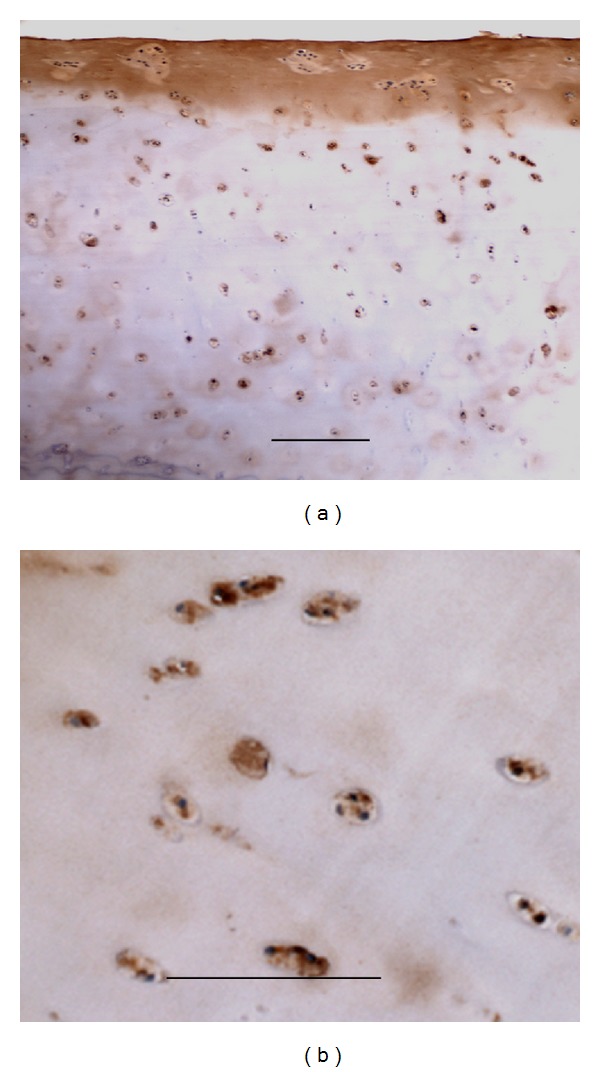
Matrilin-2 expression in total knee arthroplasty. In total knee arthroplasty samples, matrilin-2 is strongly located in extracellular matrix of the surface of joint (a) and chondrocytes of articular cartilage (b). Bar, 100 *μ*m.

Osteoarthritis (OA) is one of the most common and disabling diseases in the elderly, affecting nearly 80% of individuals older than 75 years [30, 31]. OA is due to wear and tear with the loss of articular cartilage. Articular cartilage degeneration and insufficient self-repair were believed to be the primary cause of OA [30]. In this study, an OA developmental animal model of Duncan-Hartley guinea pigs samples was investigated by Goldner staining and immunohistochemistry; the guinea pigs and clinic knee arthroplasty samples were studied by immunohistochemistry. The Goldner staining for the tibiae of Duncan-Hartley guinea pigs showed remarkable damage in articular cartilage (Figures 5(a) and 5(b)). The results of immunohistochemistry showed strong expression of matrilin-2 in articular cartilage including surfacing area and proliferating and hypertrophic areas in the OA developmental animal model (Figures 6(a)–6(d)). In total knee arthroplasty samples, significantly increased expression level of matrilin-2 was observed in articular cartilage (Figures 7(a) and 7(b)).

## 4. Discussion

Matrilins are of multiple domain proteins [1, 5–7, 20]. The members of this family are involved in the formation of filamentous networks in the extracellular matrices of various tissues [1, 5–7, 20]. Based on mouse matrilin-2 sequence, matrilin-2 precursor with 956 amino acid residues consists of a N-terminal putative signal peptide, two von Willebrand factor type A-like domains (vWFA) connected by 10 epidermal growth factor-like (EGF) repeats, one unique domain, and a series of heptad repeats at its C-terminal coiled-coil domain; and its predicted molecular mass is approximately 106.8 kDa [9, 20]. Because both vWFA domain and EGF domain have been identified in ubiquitously extracellular matrix and cell membrane and nucleus which participate in cell adhesion, protein-protein interaction, and the formation of multiprotein complexes [5, 6, 32–34], the antibody raised by whole-length recombinant protein may usually have cross reactions with other molecules. Matrilin-2 contains a unique domain located between the second vWFA domain and the coiled-coil domain [1, 9, 20], which provides us with an opportunity to raise matrilin-2-specific antibody by using this region as an antigen. In the present work, we generated a specific antibody to recognize the unique domain of matrilin-2, which is an ideal reagent to investigate the distribution pattern of matrilin-2 in immunohistochemical analysis. The results of Western blotting analysis confirm that the antibody raised in the present study works well for both long and short forms of matrilin-2 but not for deleted mutant of matrilin-2. Importantly, the antibody does not have cross reactions with matrilin-1 and matrilin-3.

The matrilin family contains four members, that is, matrilins 1, 2, 3, and 4. Matrilin-1 and matrilin-3 are cartilage-specific proteins, and their biological functions have widely been investigated. Matrilin-1 is involved in a variety of inherited chondrodysplasia [35–37]. Three microsatellite polymorphisms in this gene were associated with idiopathic scoliosis [37, 38]. Matrilin-1-specific antibody and complement activation could mediate relapsing polychondritis [39]. Matrilin-3 is the smallest member in matrilin family [40]. Mutations of matrilin-3 have been reported in a variety of skeletal diseases, including multiple epiphyseal dysplasia which is characterized by irregular ossification of the epiphyses and early-onset osteoarthritis, spondylo-epi-metaphyseal dysplasia, and idiopathic hand osteoarthritis [27, 31, 41–49]. Matrilin-2 and matrilin-4 are found to be widely distributed in many organs but we know a little about their functions. In this study, we examined the distribution pattern of matrilin-2 and found that there is heterogeneity of matrilin-2 expression in each different tissue.

Although Klatt et al. [50] have observed the expression of matrilin-2 in skeletal tissues of embryo at day 14.25 p.c. and newborn and 6-week-old mice by immunohistochemical analysis, the expression of matrilin-2 in nonskeletal tissues is unknown. Our results showed that matrilin-2 expression occurs in the cartilage of newborn mouse tibia including resting, proliferating, hypertrophic zone, and perichondrium, periosteum, bone marrow, and ligaments in the newborn mice. The strongest expression is in hypertrophic chondrocytes. The immunostaining signal in the present study showed much stronger than that of Klatt's observation. In adult mice, matrilin-2 expression level is much decreased. The expression mainly occurs in articular cartilage of surface area and hypertrophic chondrocytes. There are no positive signals detectable in other zones of cartilage and bone marrow. Our results are compatible with Klatt's results in adult mice. By quantitative real-time RT-PCR and Western blotting analyses, the mRNA and protein levels of matrilin-2 showed to be significantly decreased in the adult mice compared to the newborn mice. These results are consistent with the observation by immunohistochemistry. Therefore, matrilin-2 may be age-dependent expression in skeletal system.

In the nonskeletal tissues, the expression of matrilin-2 mainly occurs around cardiac muscle cells and Purkinje fibers in the heart; around corpus spongiosum in the penis; submucosa in the colon and esophagus; extracellular matrix of cartilage in the tracheal cartilage; and, glomerulus, the basement membrane of distal convoluted tubule and renal matrix in kidney. The observations of this study demonstrate that matrilin-2 expressed in skeleton, dense and loose connective tissues, and some specialized epithelia in mice, which consists of a large filamentous network in the body. Furthermore, matrilin-2 acts as an adapter molecule connecting other proteins and proteoglycans in the extracellular matrix and plays an important role in the communication or balance between the extracellular matrix and epithelial cells.

Osteoarthritis (OA) is affecting nearly 80% of individuals older than 75 years and is one of the most common and disabling diseases in the senior population [30, 31]. OA is due to wear and tear with loss of articular cartilage [8, 30, 31]. Cartilage degeneration and insufficient self-repair were believed to be the primary cause of OA [28, 30]. In this study, an OA developmental animal model of Duncan-Hartley guinea pigs and clinic total knee arthroplasty samples was investigated by Goldner trichrome staining and immunohistochemistry. The Goldner trichrome staining for the tibiae of Duncan-Hartley guinea pigs revealed the damage in articular cartilage. By immunohistochemistry, matrilin-2 was strongly expressed in articular cartilage including surfacing area and proliferating and hypertrophic areas. In total knee arthroplasty samples, matrilin-2 was also highly expressed in articular cartilage. The observation demonstrates that the expression of matrilin-2 may be used as a potential biomarker of osteoarthritis in articular cartilage.

In summary, we have generated a unique domain antibody of matrilin-2 that can specifically recognize both long and short forms of matrilin-2 without cross reaction with matrilin-1 and matrilin-3. With this unique antibody, we examined the pattern of matrilin-2 expression in skeletal and nonskeletal tissues and found that matrilin-2 was widely expressed in many tissues and organs that may hint biological functional heterogeneity of matrilin-2. Moreover, we found that matrilin-2 may be a potential early biomarker of OA developmental articular cartilage.

## Abbreviations

RT-PCR: Reverse transcription-polymerase chain reaction
ECM: Extracelluar matrix
vWFA: von Willebrand factor A
EGF: Epidermal growth factor
DCT: Dense connective tissue
LCT: Loose connective tissue
CMP: Cartilage matrix protein
OA: Osteoarthritis.
